# Cost-Effective Data Acquisition Systems for Advanced Structural Health Monitoring

**DOI:** 10.3390/s24134269

**Published:** 2024-06-30

**Authors:** Kamer Özdemir, Ahu Kömeç Mutlu

**Affiliations:** Civil Engineering Department, Gebze Technical University, 41400 Kocaeli, Turkey; k.ozdemir2020@gtu.edu.tr

**Keywords:** MEMS-based Sensors, geophone sensors, Raspberry Pi, structural vibration, weak motion—strong ground motion measurements, cost-effective device design, Python

## Abstract

With the growing demand for infrastructure and transportation facilities, the need for advanced structural health monitoring (SHM) systems is critical. This study introduces two innovative, cost-effective, standalone, and open-source data acquisition devices designed to enhance SHM through the latest sensing technologies. The first device, termed CEDAS_acc, integrates the ADXL355 MEMS accelerometer with a RaspberryPi mini-computer, ideal for measuring strong ground motions and assessing structural modal properties during forced vibration tests and structural monitoring of mid-rise buildings. The second device, CEDAS_geo, incorporates the SM24 geophone sensor with a Raspberry Pi, designed for weak ground motion measurements, making it suitable for seismograph networks, seismological research, and early warning systems. Both devices function as acceleration/velocity Data Acquisition Systems (DAS) and standalone data loggers, featuring hardware components such as a single-board mini-computer, sensors, Analog-to-Digital Converters (ADCs), and micro-SD cards housed in protective casings. The CEDAS_acc includes a triaxial MEMS accelerometer with three ADCs, while the CEDAS_geo uses horizontal and vertical geophone elements with an ADC board. To validate these devices, rigorous tests were conducted. Offset Test, conducted by placing the sensor on a leveled flat surface in six orientations, demonstrating the accelerometer’s ability to provide accurate measurements using gravity as a reference; Frequency Response Test, performed at the Gebze Technical University Earthquake and Structure Laboratory (GTU-ESL), comparing the devices’ responses to the GURALP-5TDE reference sensor, with CEDAS_acc evaluated on a shaking table and CEDAS_geo’s performance assessed using ambient vibration records; and Noise Test, executed in a low-noise rural area to determine the intrinsic noise of CEDAS_geo, showing its capability to capture vibrations lower than ambient noise levels. Further field tests were conducted on a 10-story reinforced concrete building in Gaziantep, Turkey, instrumented with 8 CEDAS_acc and 1 CEDAS_geo devices. The building’s response to a magnitude 3.2 earthquake and ambient vibrations was analyzed, comparing results to the GURALP-5TDE reference sensors and demonstrating the devices’ accuracy in capturing peak accelerations and modal frequencies with minimal deviations. The study also introduced the Record Analyzer (RECANA) web application for managing data analysis on CEDAS devices, supporting various data formats, and providing tools for filtering, calibrating, and exporting data. This comprehensive study presents valuable, practical solutions for SHM, enhancing accessibility, reliability, and efficiency in structural and seismic monitoring applications and offering robust alternatives to traditional, costlier systems.

## 1. Introduction

Micro-Electro-Mechanical Systems (MEMS) sensor technology has been undergoing rapid development for several decades. These sensors, known for their exceptional sensitivity, have garnered significant attention. The cost-effectiveness, small form, lightweight nature, and minimal power consumption of MEMS sensors render them well-suited for addressing the diverse challenges inherent to specific application environments. Examples of how such environments are specific include automotive crash detection and tire pressure monitoring, industrial predictive maintenance, precision manufacturing, navigation in drones and UAVs, balance control in robotics, spacecraft and satellite monitoring in space exploration, astronaut health monitoring, and structural health monitoring during earthquakes. These environments present challenges such as extreme conditions, precision requirements, and power efficiency needs, where MEMS sensors excel. Over time, MEMS accelerometers have found extensive utility across diverse fields, with widespread adoption in sectors such as automotive, machinery, navigation systems, robotics, and human space exploration [1,2]. In response to the evolving demands of these domains, these sensors have evolved to become more compact, sensitive, power-efficient, and ideally suited for earthquake monitoring applications [3]. Vibration sensors, in particular, have seen widespread use in space and automotive electronics and are now gaining traction for their exceptional precision in applications such as seismic and gravity measurements, navigation systems for autonomous vehicles and pedestrians, and the development of portable high-precision MEMS-based gravimeters and seismometers. The pioneering demonstration of MEMS accelerometers dates back to 1979 at Stanford University, where an accelerometer device was fabricated in a compact 2 × 3 × 0.6 mm package, weighing a mere 0.02 g. This device exhibited the capability to measure accelerations as small as 10 mg over a 100 Hz bandwidth, with a measurement range extending up to 50 g. This marked the inception of integrated-circuit fabrication for accelerometers, representing one of the earliest applications of MEMS accelerometers, well before their widespread adoption, as we witness today [4].

The Structural Health Monitoring (SHM) and Earthquake Observation (EO) systems have witnessed substantial improvements in recent years, driven by the miniaturization, heightened sensitivity, and enhanced data quality offered by MEMS sensors. Especially in SHM applications, while conventional simulation techniques and simplified design approaches are frequently employed to characterize structures’ mechanical attributes, a comprehensive evaluation of the actual structural behavior across various structural typologies is limited [5]. Traditional systems were disadvantageous by their exorbitant costs and the logistical challenges associated with maintaining monitoring systems over extended periods of time. Nevertheless, MEMS technology has effectively surmounted these constraints, rendering SHM and EO systems cost-effective and facilitating their deployment in large-scale applications [6].

The ANSS (Advanced National Seismic System) instrumentation guideline, for instance, provides detailed instructions for structural monitoring and the establishment of monitoring network stations at national, regional, and urban scales. This guideline categorizes instruments (such as MEMS-based accelerometers and geophone devices) and offers expected performance specifications for the recommended instrument depending on the specific application [7,8]. Additionally, [9] have developed guidelines encompassing performance testing procedures for weak motion velocity sensors and strong-motion accelerometers. According to [9], weak motion sensors, such as broadband velocity seismometers, are designed to detect low-amplitude seismic waves typically occurring during minor earthquakes or ambient noise, making them highly sensitive to subtle ground motions. These sensors generally have a lower clip level and can measure ground velocities in the range of micro-meters per second (µm/s). On the other hand, strong-motion accelerometers are engineered to record higher-amplitude seismic waves that occur during moderate to large earthquakes. These devices can handle more intense ground motions without saturating and typically measure ground accelerations in the range of several meters per second squared (m/s^2^), with peak values reaching up to tens of m/s^2^ [9]. Ref. [10] explored the viability of low-cost MEMS accelerometers commonly found in mobile phones and laptops for applications in strong motion seismology. Low sensitivity in MEMS sensors generally refers to their ability to detect small ground motions and is often quantified in terms of their minimum detectable acceleration. Typically, this value ranges from a few tens to hundreds of micro-g (µg), where 1 g is the acceleration due to gravity (9.8 m/s^2^). High noise, on the other hand, is characterized by the noise density of the sensors, which is a measure of the sensor’s intrinsic electronic noise. For MEMS accelerometers, this noise density is usually in the range of tens to hundreds of micro-g per square root hertz (µg/√Hz). The tested devices exhibited low sensitivity and high noise density, rendering them suitable only for near-field earthquakes with a magnitude of 5 or higher [10]. Involving citizens, utilizing low-cost MEMS sensors (refers to sensors that are significantly cheaper than traditional seismic sensors such as broadband seismometers and high-end accelerometers. Traditional broadband seismometers, which are highly sensitive and used in professional seismic monitoring, can cost several thousand to tens of thousands of dollars), and deploying networks within urban areas aligns with the broader objectives of enhancing earthquake understanding and bolstering early warning systems to fortify infrastructure resilience. Initiatives such as the Community Seismic Network, QuakeCatcherNetwork (QCN), Urban Seismic Network, Self-organizing Seismic Early Warning Information Network (SOSEWIN), and ShakeNet are noteworthy contributions to the field of seismic monitoring and earthquake research [11,12,13,14,15,16]. Some of these networks opt for user-friendly sensor solutions, such as QCN, with Phidget and O-Navi brands exemplifying these accessible and versatile options [17,18,19]. Evans et al. (2014) systematically tested multiple MEMS sensors, including those from the Phidget brand, following established guidelines [20]. [21] conducted a comprehensive review of the application of wireless MEMS-based accelerometer sensor boards for structural vibration monitoring. The article details various types of structures where these sensors are deployed, including bridges, buildings, and pipelines. These sensor boards are particularly valuable for monitoring the dynamic responses of large civil structures. For example, they are used to measure vibrations in pedestrian bridges and heritage buildings to assess their structural integrity and detect potential issues such as damage or stress accumulation. Sabato et al. (2016) highlight the advantages of these wireless MEMS sensors, including their superior noise density and resolution, which are crucial for capturing detailed vibration data [21]. Ambrož (2017) presented an illustrative example of using a Raspberry Pi single-board computer as a low-cost data acquisition system, particularly applicable for measuring acceleration, velocity, and displacement on human-powered vehicles [22]. Additionally, the Raspberry Shake device integrates both MEMS accelerometers and geophone elements, using a Raspberry computer as its processor [23]. [24] conducted tests involving multiple low-cost MEMS sensor boards on building models. These boards possessed a maximum resolution of 16 bits, but the limiting factor for these sensors was the presence of high self-noise. Consequently, the tested sensor boards failed to detect the modal frequencies of the building model under ambient vibration conditions [24]. Consequently, these budget-friendly boards, equipped with low-resolution Analog-to-Digital Converters (ADC), find suitability primarily in forced vibration scenarios, local intense ground motion studies, and educational applications [25]. Low-cost acceleration Data Acquisition Systems (DAS) have gained significant popularity within the realms of Earth Science and Earthquake Engineering studies. One of the recent studies published by Özcebe et al. (2022) delved into the utilization of the Raspberry Shake RS4D for dynamic structural identification [26].

In this study, an accelerometer was designed for strong ground motion measurements by integrating the ADXL355 sensor into the Raspberry Pi mini-computer, alongside a seismometer, achieved through the integration of the Raspberry Pi mini-computer with the SM24 geophone sensor for weak ground motion measurements. These devices are designed to be versatile and applicable in various scenarios. The Cost-Effective Data Acquisition System (CEDAS) accelerometer (CEDAS_acc) enables the evaluation of structural modal properties. It is well-suited for forced vibration tests on shake table experiments and is also appropriate for structural monitoring of mid-rise buildings. On the other hand, CEDAS_geophone devices (CEDAS_geo) form the foundational component of seismograph networks, facilitating the recording of ground motion waveforms for seismological research and early warning systems, with their sensitivity to ambient vibrations and weak ground motion.

## 2. Device Fabrication and Features

**Figure 1:**
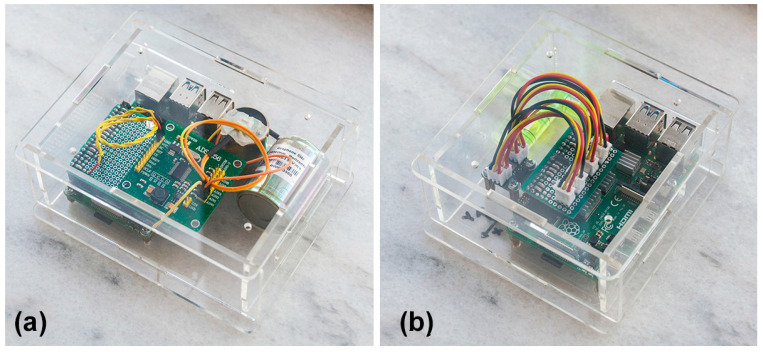
CEDAS devises photos are depicted. (**a**) CEDAS_geo device (**b**) CEDAS_acc device.

CEDAS devices function as acceleration/velocity Data Acquisition Systems (DAS) and standalone data loggers, featuring both hardware and software components. The hardware of these designed CEDAS devices comprises a single-board mini-computer, a sensor (ADXL355 MEMS sensor), an Analog-to-Digital Converter (ADC), and a micro-SD card. These components are housed within a protective casing (plexiglass material 25 × 25 × 8 cm with a 3 mm wall thickness) with a flat surface, as illustrated in Figure 1. Specifically, the CEDAS_acc incorporates a triaxial MEMS accelerometer equipped with three ADCs on the sensor board. Conversely, the CEDAS_geophone device utilizes both horizontal and vertical geophone elements, paired with an ADC board. Both are powered by 220 volt electricity.

**Figure 2:**
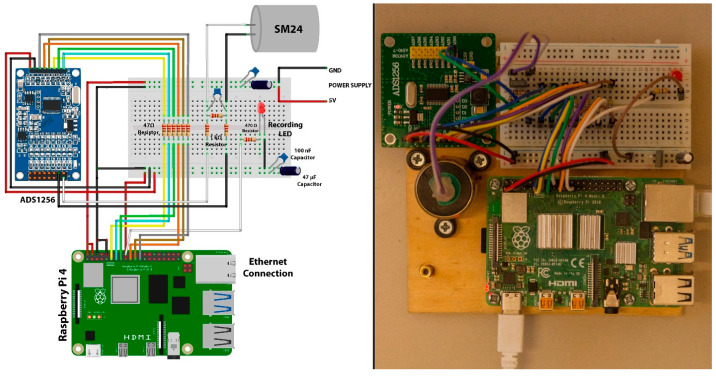
ADS1256—SM24 connection diagram.

While the devices can be operated by connecting a keyboard and monitor directly, they also offer the flexibility of remote connectivity. Users can utilize an Ethernet port via an RJ45 cable or establish a wireless connection (the Raspberry Pi has built-in Wi-Fi support) to connect to the CEDAS devices from another computer. Using a Raspberry Pi’s WiFi capabilities, users can establish wireless connections for various applications, including data acquisition, remote monitoring, and control systems. This can be achieved through SSH for secure remote command line access, VNC for remote desktop access, MQTT for lightweight messaging in IoT applications, and HTTP/HTTPS for web-based data transmission. Security features available for these configurations include password and public key authentication for SSH, SSL/TLS encryption for HTTPS and MQTT, WPA2/WPA3 encryption for WiFi networks, and access control measures such as firewalls, VPNs for secure remote connections, and MAC address filtering. Additionally, disabling unused services can minimize potential security risks. Implementing these measures ensures that data remains secure and that the system is protected against unauthorized access and eavesdropping. The communication between the single-board computer (Raspberry Pi) and the sensor board, as well as the ADC, occurs through Serial Peripheral Interface (SPI) serial communication. SPI drivers for both devices are developed using the Python 3.12.3 programming language, adhering to the specifications outlined in the device datasheets [27,28]. Additionally, the GPIO pinouts for the Raspberry Pi 4 Model B and the pinouts for the CEDAS devices’ sensor and ADC boards are configured by using datasheets [27,28,29]. A geophone is a sensor that outputs analog voltage according to vibration. SM-24 is a geophone element with a bandwidth of 10–240 Hz, generally used in seismic surveys. To connect this sensor to the Raspberry Pi, an analog-to-digital converter must be used. In the circuit, a 24-bit, low-noise delta-sigma converter ADS1256 is used. The connection diagram of the converter using the SPI communication protocol is given in Figure 2. In the schematic, the Raspberry computer is powered from the 5 V GPIO pin, and in the next photo, it is powered from the battery and Type-C port.

**Figure 3:**
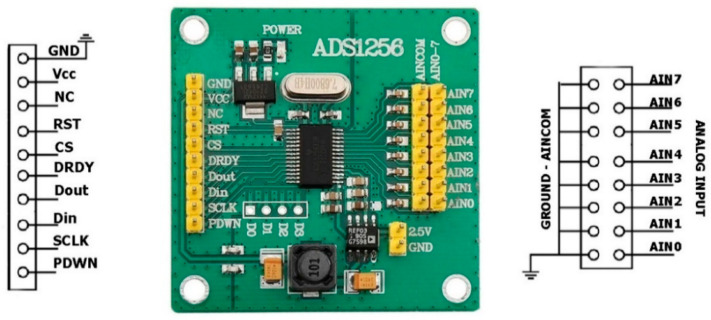
CEDAS_geo ADC board header pinout [27].

**Figure 4:**
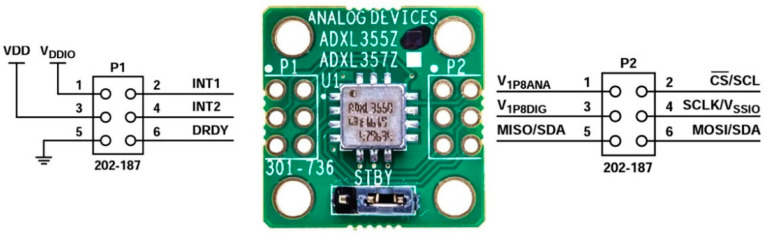
CEDAS_acc sensor board header pinout [28].

**Figure 5:**
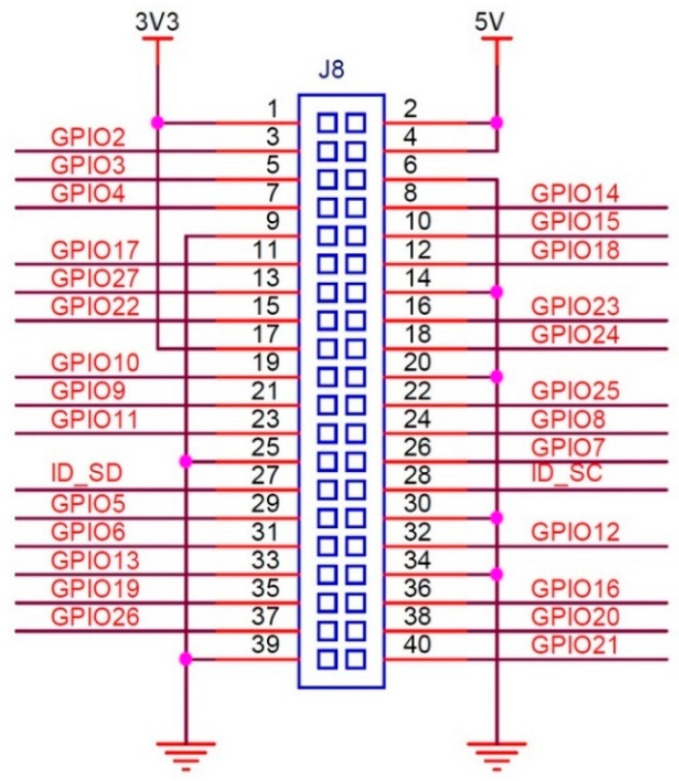
Raspberry Pi 4 Model B GPIO connector pinout [29].

Raspberry Pi 4 Model B GPIO pinout and CEDAS device sensor and ADC board pinouts are shown in Figure 3, Figure 4 and Figure 5.

Table 1 and Table 2 provide details on the SPI serial communication connections between the slave MEMS accelerometer board and ADC board tand the master Raspberry Pi computer.

**Figure 6:**
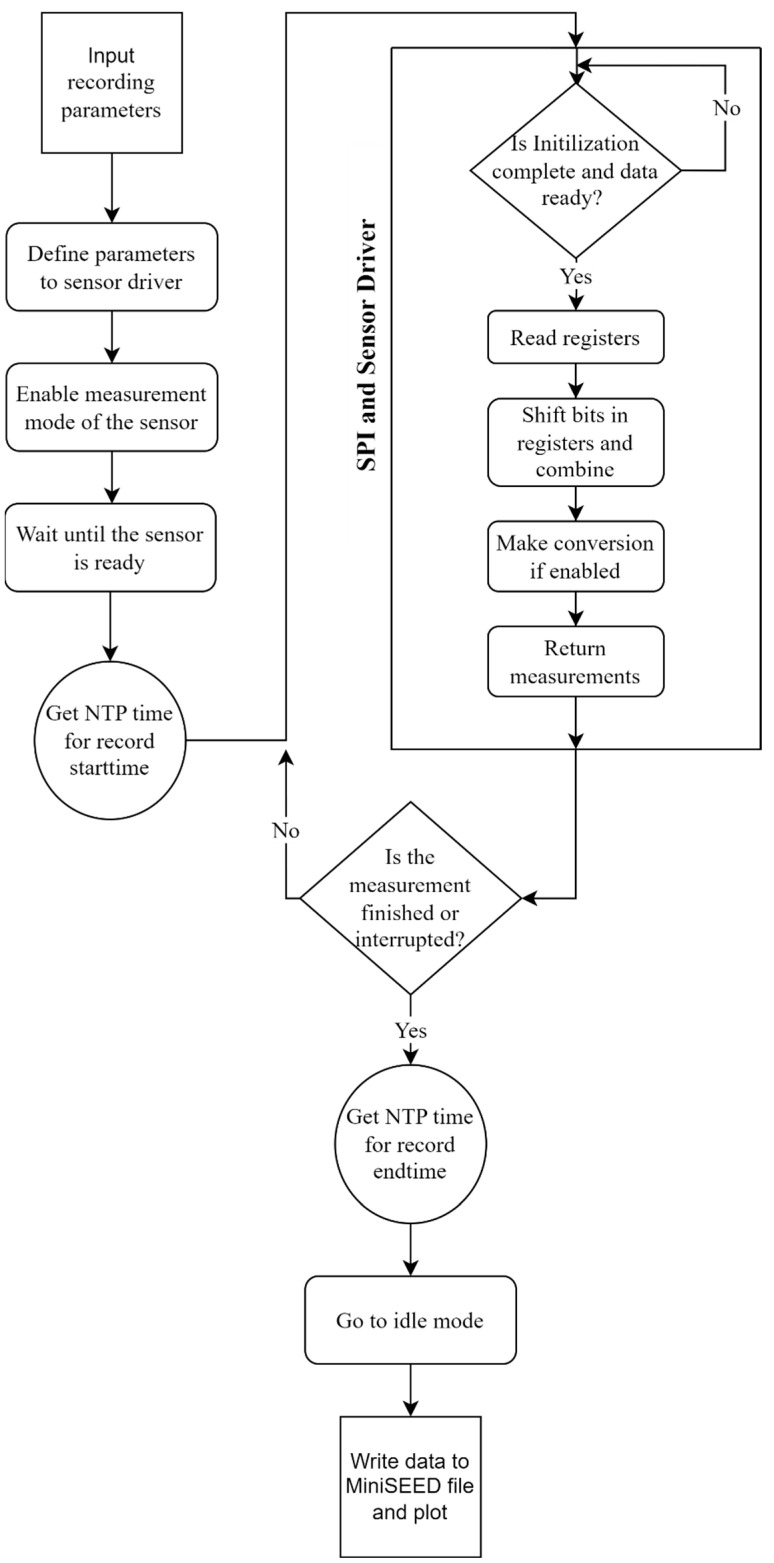
CEDAS device algorithm flowchart.

To ensure effective and precise control of both the ADXL355 MEMS sensor and ADS1256 ADC, a meticulously designed algorithm is used for the Serial Peripheral Interface (SPI) and sensor drivers. The algorithm flowchart for the drivers of both CEDAS_acc and CEDAS_geo devices share a similar structure with minor modifications to accommodate the specific characteristics of each device and the systematic steps involved in controlling and processing data from the ADXL355 MEMS sensor and ADS1256 ADC (Figure 6).

The algorithm begins with user input, requiring the configuration of sensor parameters and the header information for the MiniSEED file. Users must specify the sampling rate, number of data points, and details such as the network, station, location, and channel names. The start and end times of the records are automatically added to the headers in UTC datetime format. The MEMS sensor driver is then configured with the defined measurement range and sampling rate. For the CEDAS_geo device, parameters such as sampling rate, input channel, and programmable gain amplifier (PGA) value are set for the ADC driver.

Once the parameters are configured, the algorithm restarts the conversion cycle and activates the measurement mode via the driver. A delay of 50 milliseconds is incorporated to account for the devices’ settling time, which is adequate for both devices. Prior to initiating the recording loop, the device activates the recording light and acquires the NTP time to mark the start time of the measurement. Within the recording loop, the algorithm enters another polling loop to monitor the Data Ready (DRDY) pin of the sensor and ADC. The ADC operates in continuous mode as specified in the datasheet, enabling data reading without waiting or synchronizing for the conversion cycle to restart.

When new measurement data is available, the polling loop terminates, and the data is read from the registers. Each device retrieves three bytes via the SPI bus, with the MEMS sensor providing 20-bit data for each axis and the ADC delivering 24 bits. These bytes are combined and converted to integer values. Depending on user input, the raw data is converted into acceleration or velocity. Finally, the data is returned and stored in an array. If the recording duration has not concluded and there is no interruption, the recording loop persists. Otherwise, the algorithm records the measurement end time in UTC datetime, transitions the device to idle or low power mode, and writes the recorded data from the array to MiniSEED format along with the header information.

## 3. Description of Data Storage and Processing

CEDAS devices are equipped to store recorded acceleration and velocity data on an integrated micro-SD card. For optimal performance, it is recommended to use an SD card with a minimum capacity of 8 gigabytes and the capability to write at a minimum speed of 10 megabytes per second. These capacity specifications ensure that the micro-SD card has sufficient space and writing speed to store the data generated by the CEDAS devices during their standard operational conditions. The recorded data from CEDAS devices is stored in a MSEED format file. The comprehensive set of information in the file header ensures that the stored MSEED format file contains the necessary metadata to accurately interpret and analyze the waveform data recorded by the CEDAS devices.

Data processing on CEDAS devices involves the conversion of raw data, filtering, time domain analysis and frequency domain analysis. The conversion of raw data to a desired unit of acceleration or velocity depends on the hardware of the device and user-selected inputs. The formula of the raw data to velocity conversion factor $K_{conversion}$ for the CEDAS_geo is given below:

(1) $$K_{conversion}=\frac{2\times ReferenceVoltage}{Geophonesensitivity\times ADCGain\times ADCbitcount}$$

In this study, a 5 Hz horizontal geophone element with 80 V/m/s sensitivity, 24-bit ADC ,and PGA parameters set to 16. The reference voltage of the ADC board is 2.5 V. Raw data is converted to a velocity unit of m/s with a factor calculated below:

(2) $$K_{conversion}=\frac{2\times 2.5V}{80\frac{V}{m/s}\times 16\times 2^{24}}=2.328\times 10^{-10}m/s$$

CEDAS_acc has a default ±2.048 g measurement range with a 20-bit ADC. Raw sensor data can be converted to an acceleration unit of g by multiplying it with the given value below:

(3) $$K_{conversion}=\frac{TotalRange}{ADCbitcount}=\frac{2\times 2.048g}{2^{20}}=3.9\times 10^{-6}g$$

In this study, throughout the conducted tests, low-pass Butterworth filters were used [30]. Depending on the analysis, different window sizes are used for spectral analysis in the following sections. Windows are averaged by 50% overlap with Hanning windows. The Tukey type window is used in spectrogram calculations to maintain statistical independence between segments compared to Welch’s method.

The Record Analyzer (RECANA) web application offers a user-friendly interface for managing data analysis on CEDAS devices. Accessible via web browsers, RECANA supports MSEED, SAC, or GCF formats. It comprises two main sections: an Import Section for uploading and viewing data, where time series are automatically generated from header information, and a Filter and Export Section for further data processing. Users can calibrate data by inputting calibration factors and units, and trim time series using a slider element. The Filter and Export Section includes features for detrending, designing and applying filter kernels, and exporting data in various formats. RECANA simplifies the data analysis process for CEDAS devices, providing flexibility and efficiency for users in importing, visualizing, calibrating, trimming, filtering, and exporting data.

## 4. Validation Tests and Case Study

The assessment of CEDAS_acc and CEDAS_geo devices encompass three distinct evaluations: an accelerometer offset test, an ambient vibration test to ascertain the frequency response of the geophone device, and a high-level excitation test employing a shake table to discern the frequency response and linearity of the accelerometer device. Throughout these assessments, the GURALP-5TDE strong motion accelerometer serves as a benchmark reference device. Measurements conducted during the tests maintain a ±2 g measurement range and 125 SPS for CEDAS_acc and a ±1.6 mm/s measurement range with PGA set to 16 and 100 SPS for CEDAS_geo devices, unless explicitly stated otherwise. In contrast to CEDAS_acc and GURALP-5TDE, velocity records of CEDAS_geo are converted to acceleration prior to subsequent analyses.

### 4.1. Offset Test

**Figure 7:**
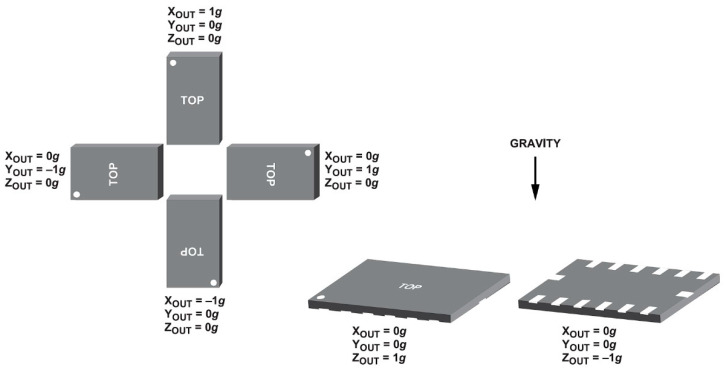
Sensor orientations used in the offset calibration test [19].

Offset, or bias, represents the deviation of a sensor’s output from the true or expected value when the input should be zero. It is crucial to compensate for and correct this offset to ensure accurate and reliable measurements, especially in compliance with regulations. Offset refers to the DC (0 Hz) output level of the sensor when no motion is acting on it. The offset test is systematically executed by affixing the sensor to a precisely rectilinear cubic box. Subsequently, the box is positioned on a meticulously leveled flat surface, and recordings are obtained for six possible orientations [19] (Figure 7).

**Figure 8:**
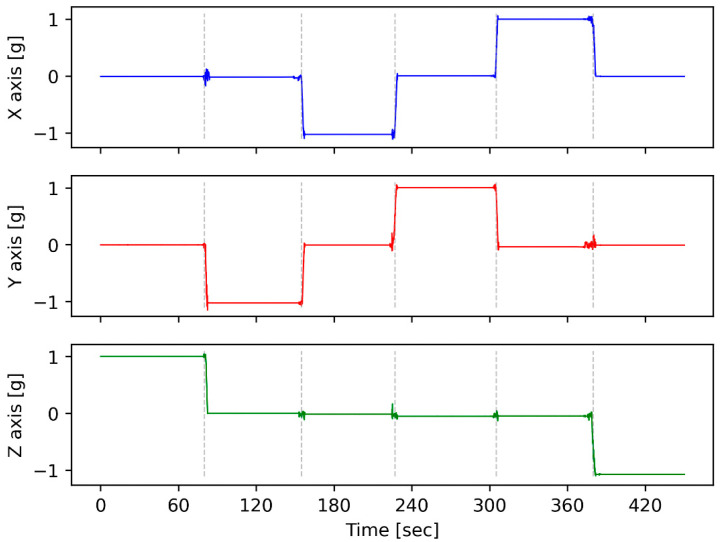
Time series from the CEDAS_acc offset test depicts a total of six orientations. Segments divided by dashed lines represent each flip.

The accelerometer incorporated into the device exhibits a flat frequency response from DC up to approximately 500 Hz [28]. This characteristic advantage allows for the assessment of the device using gravity as a reference input. Contrastingly, geophones display reduced responsiveness at frequencies below their inherent frequency. Additionally, deploying the horizontal geophone element against gravity is unfeasible. Consequently, this motionless test can solely be conducted utilizing an accelerometer device. For each orientation, 60 s of data is averaged, and offset values for each axis are calculated. Depending on the orientation, the actual measurement is equal to +1 g or −1 g. By subtracting the actual measurement from the average of 60 s of measured data, the offset value for the related axis and orientation is calculated (Figure 8).

The accuracy errors for each axis are consistently below 1%, aligning with the stringent ANSS instrumentation guideline specifications for Class A and Class B accelerometers [5]. The maximum offset is identified as 2% for the X axis and 6% for the Y axis. Notably, these values fall below the maximum offsets stipulated in the sensor datasheet [28]. Calibration of these offsets is accomplished by subtracting the measured errors from the sensor outputs. Subsequently, the calibration factor of the instrument is redefined in preparation for subsequent tests [31].

### 4.2. Frequency Response Tests

**Figure 9:**
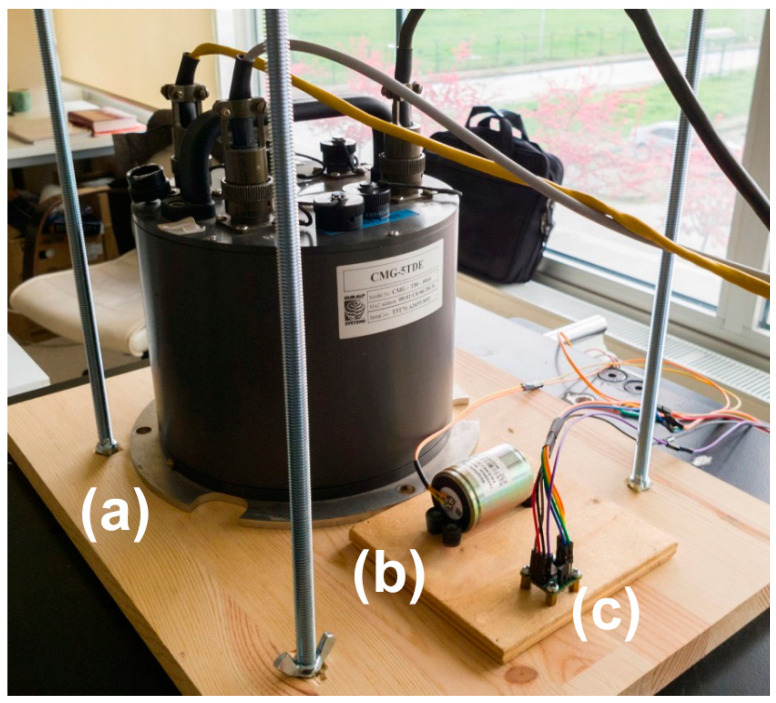
Sensor placements for frequency response tests of (**b**) geophone and (**c**) accelerometer device sensors with (**a**) GURALP-5TDE reference sensor.

Frequency response tests for CEDAS_acc and CEDAS_geo were conducted at the Gebze Technical University Earthquake and Structure Laboratory (GTU-ESL). The assessment of the geophone device involved the utilization of ambient vibration records. This examination was carried out by comparing the frequency response of the geophone device through simultaneous recordings with the GURALP-5TDE reference device and the CEDAS devices at the identical location. For the accelerometer device, the evaluation was performed on a shaking table. The process involves subjecting the accelerometer device to controlled shaking, allowing for the analysis of its frequency response under different conditions (Figure 9).

**Figure 10:**
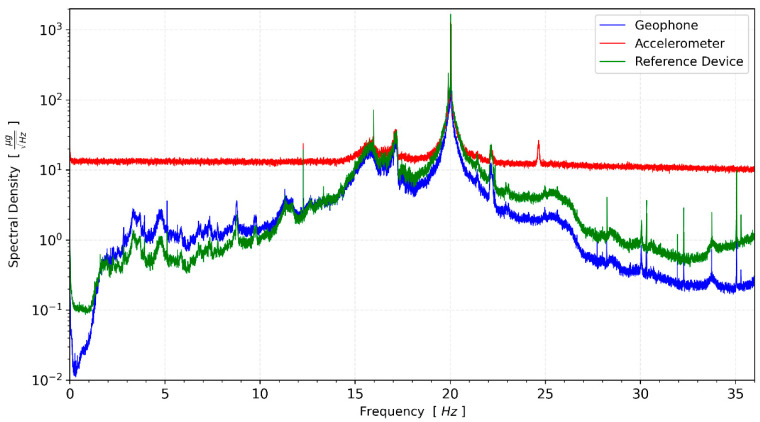
Spectral density of synchronous environmental noise recording from both devices and the reference sensor at the same location.

The CEDAS_geo, CEDAS_acc device, and GURALP-5TDE reference device captured data at the GTU-ESL for 16-h. Spectral density functions are calculated for the devices in a horizontal direction (Figure 10).

**Figure 11:**
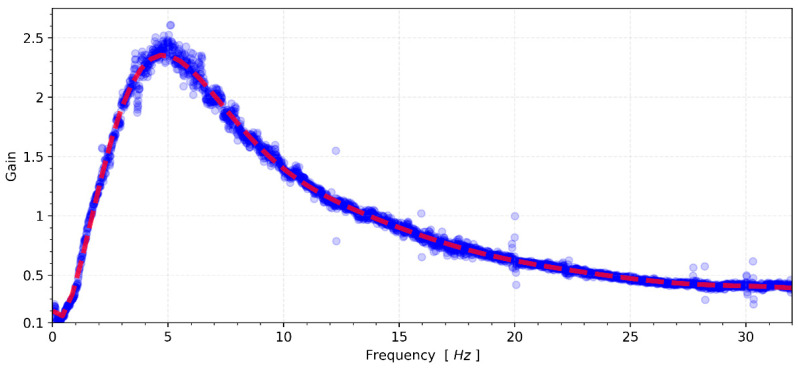
Shows the response curve (or transfer function) of the CEDAS_geo.

Compared to other sensors, the MEMS accelerometer device has a higher noise density and cannot capture signals lower than 15 μg/√Hz spectral density. Therefore, this test is not suitable to evaluate the frequency response of the accelerometer in these conditions. However, CEDAS_geo is not limited by instrumental noise and can be evaluated. The transfer function of the CEDAS_geo device is derived by utilizing the spectral density of the GURALP-5TDE reference sensor as input and considering the CEDAS_geo as the output motion, as depicted in Figure 11.

**Figure 12:**
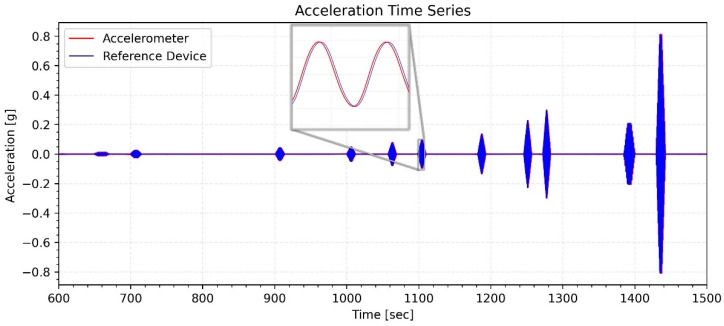
Time series of the shake tests.

**Figure 13:**
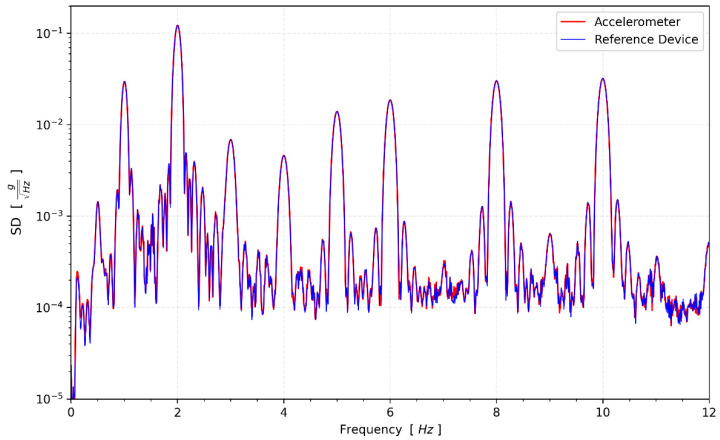
Spectral density of the shake tests.

The frequency response analysis of CEDAS_geo indicates a response exceeding the 3 dB in-band limit at frequencies proximate to the inherent frequency of 5 Hz. However, at the extremities of the frequency range, the response falls below the −3 dB in-band limit. This outcome underscores the necessity for proper signal scaling to align with the transfer function. Subsequently, in subsequent tests, the transfer function was applied to all data acquired from the CEDAS_geo to ensure accurate and reliable results. In a parallel set of tests, the frequency response of CEDAS_acc was evaluated using the GURALP-5TDE instrument as a reference sensor. These tests were conducted on a uni-axial shaking table at GTU-ESL. Each test involves the generation and simultaneous recording of five sinusoidal waves with a consistent frequency and varying amplitude (0.5 Hz, 1 Hz, 2 Hz, 3 Hz, 4 Hz, 5 Hz, 6 Hz, 8 Hz, and 10 Hz) using both the CEDAS_acc and the GURALP-5TDE devices. CEDAS_geo is excluded from these tests due to its insufficient measurement range for the shake table. Figure 12 shows the filtered time series overlays of each shake with data retrieved from the GURALP-5TDE device, and Figure 13 depicts the spectral density of the collective time series spanning all shake tests.

**Figure 14:**
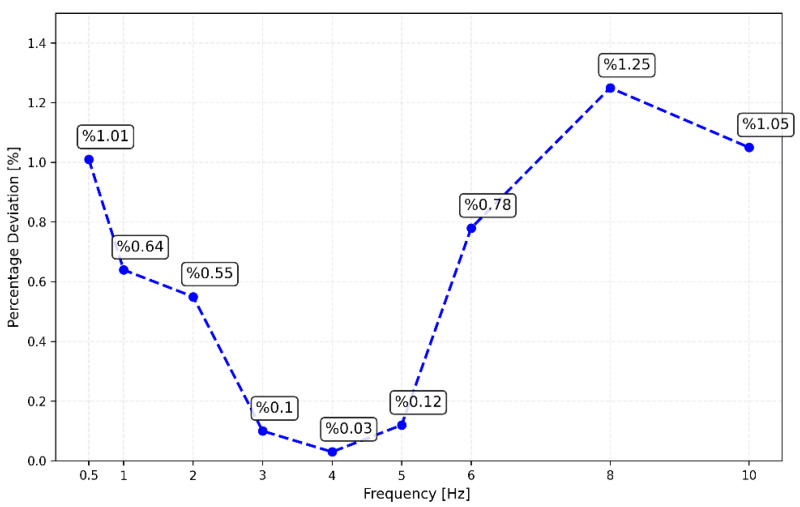
Spectral density amplitude deviation of CEDAS_acc with regard to the reference device.

The frequency values corresponding to the peak amplitudes of each shake with those of the reference sensor. This shows CEDAS_acc has an accurate and stable sampling rate. The flat response of the reference device gives an opportunity to obtain the frequency response of CEDAS_acc. Amplitude deviations between 0.5 and 10 Hz shows a maximum of 1.5% difference. Peak amplitude values and error rates for the CEDAS_acc and GURALP-5TDE devices are depicted (Figure 14). The result shows the CEDAS_acc is in the –3 dB band and has a flat response in the tested frequency range.

### 4.3. Noise Test

**Figure 15:**
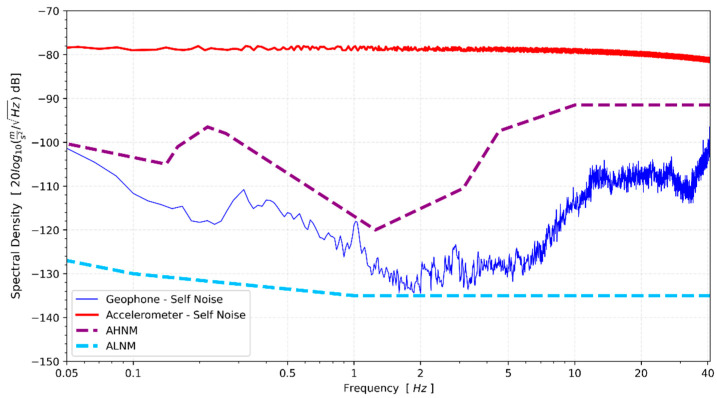
Spectral noise density of CEDAS_geo and CEDAS_acc devices in a low-noise environment compared to low-and high-noise models.

The noise test was executed at night in a rural area characterized by a low background noise level. Given the significantly higher noise level of the MEMS sensor compared to the ambient noise at the site, the purpose of this test was to ascertain the noise intensity of the CEDAS_geo. The single-sensor method was employed under the assumption that the field noise is substantially lower than the intrinsic noise of the device [32]. A 5 Hz horizontal geophone element was utilized to record the site continuously for a total duration of 45 min. The obtained results were then juxtaposed with the accelerometer high-noise model (AHNM) and accelerometer low-noise model (ALNM) developed by Cauzzi and Clinton (2013) specifically designed for accelerometers [33]. The recorded raw velocity data undergoes a conversion to acceleration, and subsequent correction of the device’s response is implemented. For CEDAS_geo, the spectral noise density was determined to fall between the low and high noise models (Figure 15). The spectral density of CEDAS_geo suggests the device’s capability to capture vibrations at levels lower than the ambient noise present at the site. However, further tests are deemed necessary to ascertain the absolute instrument noise.

### 4.4. Field Test

**Figure 16:**
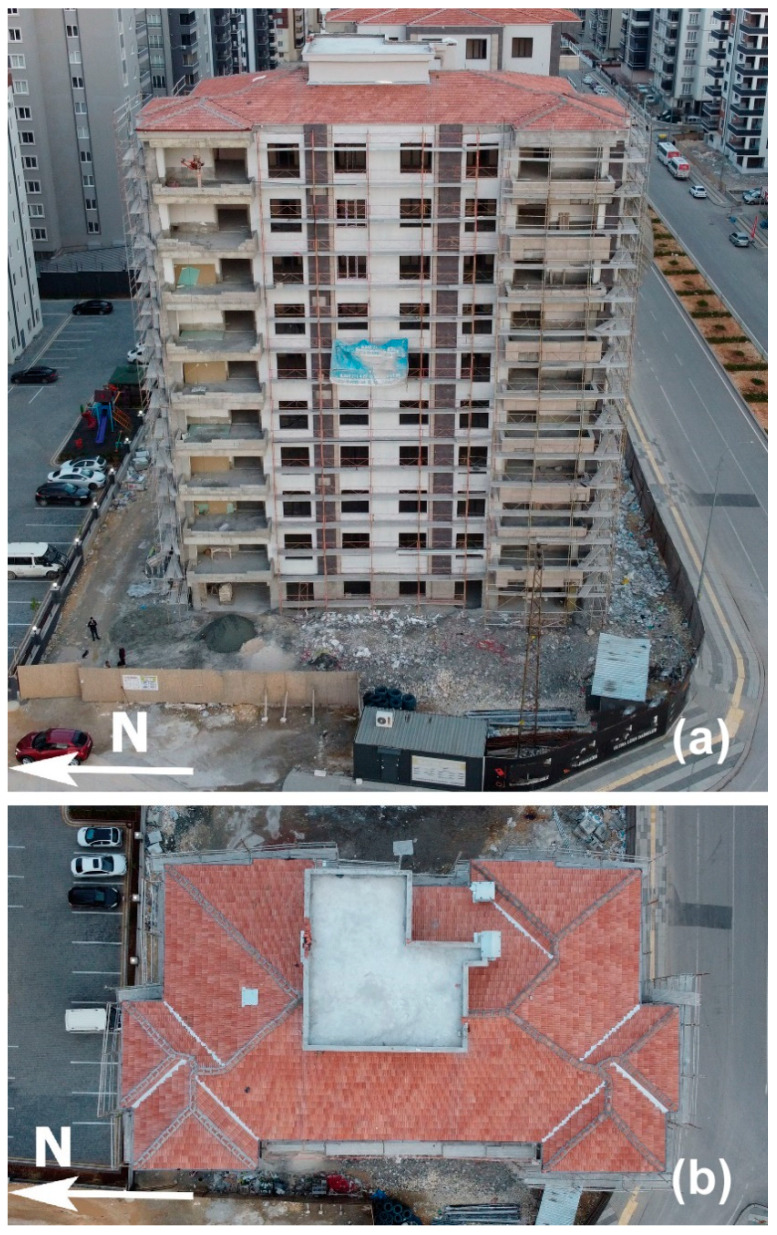
Aerial photos of the building from (**a**) side and (**b**) top.

After obtaining the characteristic parameters for the CEDAS instruments, a series of tests were carried out on a 10-story reinforced concrete building located in Gaziantep, Turkey, with temporary instrumentation conducted from 6 April 2023, to 15 April 2023 (Figure 16). The testing occurred in a region impacted by the severe earthquakes of 6 February 2023 (M7.8 and M7.6), along with subsequent aftershocks, to fully evaluate the building’s response to both earthquake vibrations and ambient conditions. The main aim of this test was to assess the performance of CEDAS instruments in real-world environmental scenarios, particularly during ground motion events affecting buildings.

**Figure 17:**
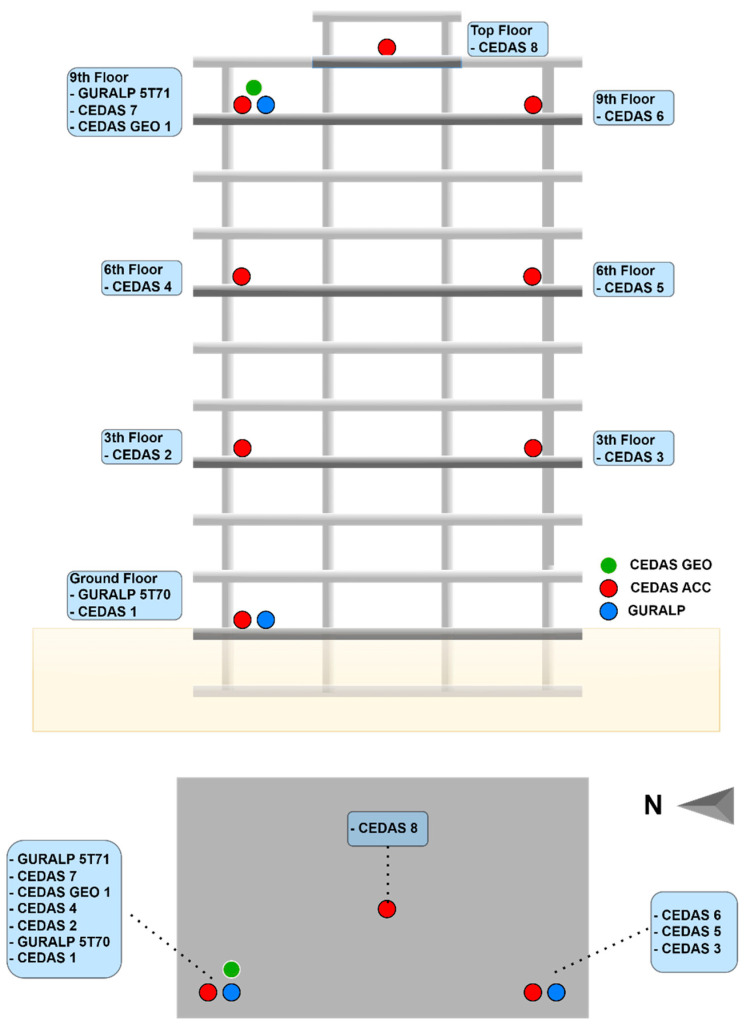
The locations of the sensors on the building.

Following guidelines outlined in codes and regulations [34], the devices were strategically placed for instrumentation. The building was equipped with 8 CEDAS_acc devices and 1 CEDAS_geo device as part of the instrumentation process (Figure 17). In addition, GURALP-5TDE strong motion accelerometers were strategically placed on both the ground floor and the 9th floor of the building to serve as reference sensors. A single CEDAS_geo device was specifically placed on the 9th floor of the building. To facilitate a direct comparison, CEDAS_acc devices were positioned next to each GURALP-5TDE accelerometer (Figure 17).

**Figure 18:**
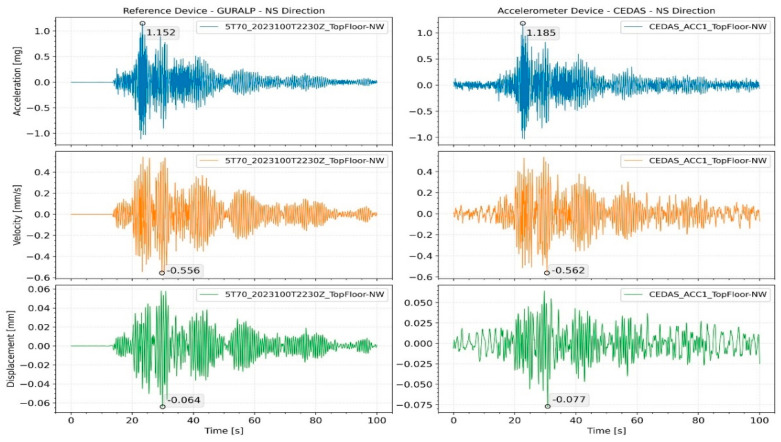
Acceleration, velocity, and displacement time series of GURALP-5TDE and CEDAS devices from the building in the NS direction.

**Figure 19:**
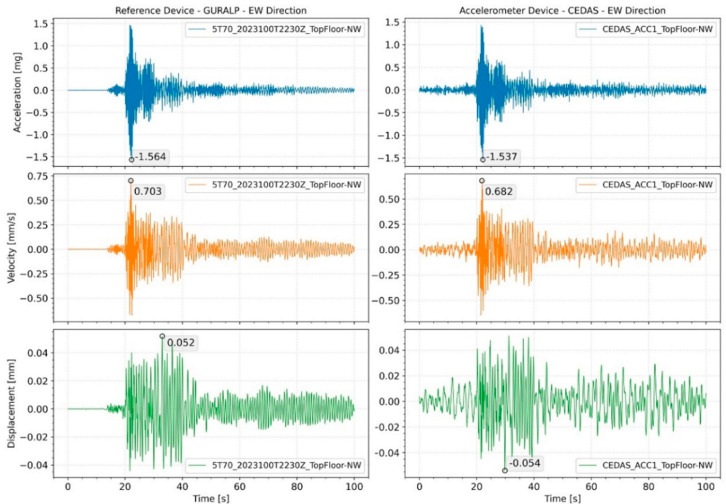
Acceleration, velocity, and displacement time series of GURALP-5TDE and CEDAS devices from the building in the EW direction.

In order to test the force vibration performance of the devices, an earthquake of magnitude M3.2 was recorded on 13 April 2023, at 03:36:05 in Gaziantep/Nurdağı (KOERI-BDTIM) with an epicenter of 39 km, and the response of the building was analyzed. For the GURALP-5TDE device, the peak acceleration at the basement floor was 0.24 mg in the NS direction and 0.4 mg in the EW direction, while for the CEDAS_acc, it was 0.25 mg in the NS direction and 0.41 mg in the EW direction. By integrating the acceleration data, the velocity and displacement time series for the devices located side by side in the basement and 9th floor are calculated and shown in Figure 18 for the NS direction and Figure 19 for the EW direction.

**Figure 20:**
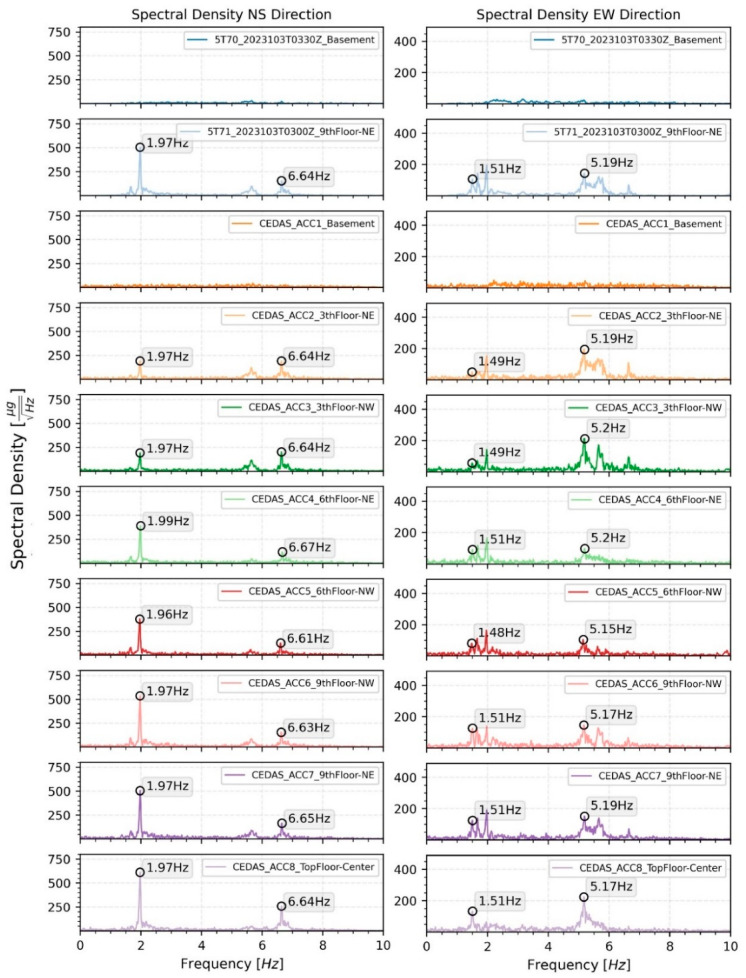
Spectral density of the response of the building in the NS and EW directions.

The calculated spectral density for all devices located on the building for both the NS and EW directions is shown in Figure 19, along with the modal frequencies of the 1st and 2nd translational modes. The response of the building during an earthquake is analyzed by the Spectral Density method. According to analysis, the 1st and 2nd translational modes were found to be 0.51 s and 0.15 s for the NS direction and 0.66 s and 0.19 s for the EW direction, respectively (Figure 20). Additionally, torsional modes are observable with 0.6 s and 0.17 s periods.

**Figure 21:**
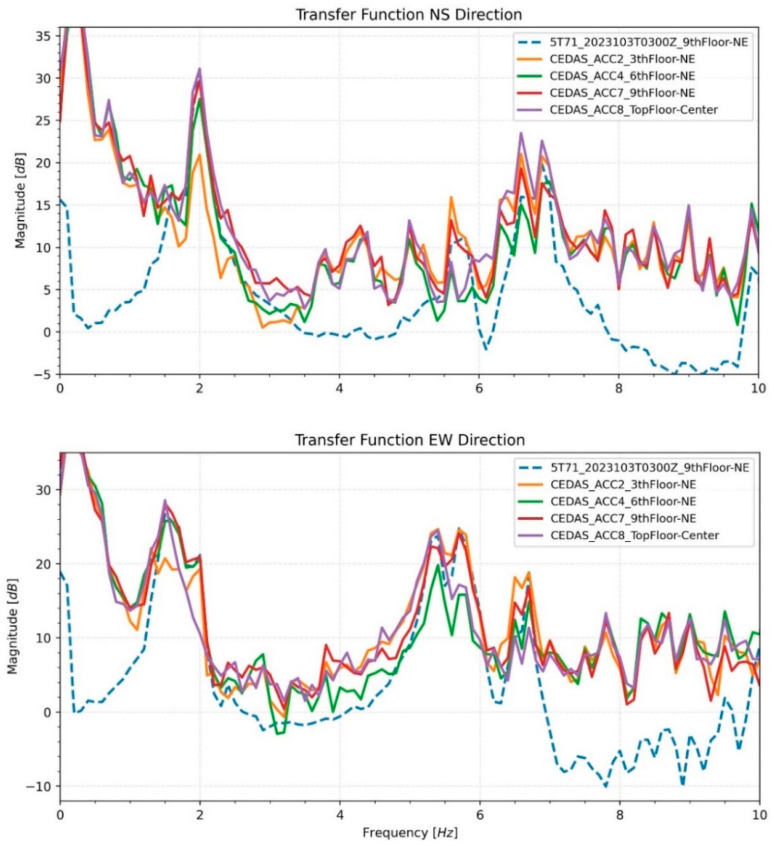
Transfer functions of devices from different floors of the building in NS and EW directions.

Transfer functions are generated by using the basement recording of the GURALP-5TDE device as an input. As an output, devices located at the 3rd, 6th, and 9th floor NE locations and the top floor center are used. Estimated transfer functions are shown in Figure 21 for the NS and EW directions of recordings, respectively.

**Figure 22:**
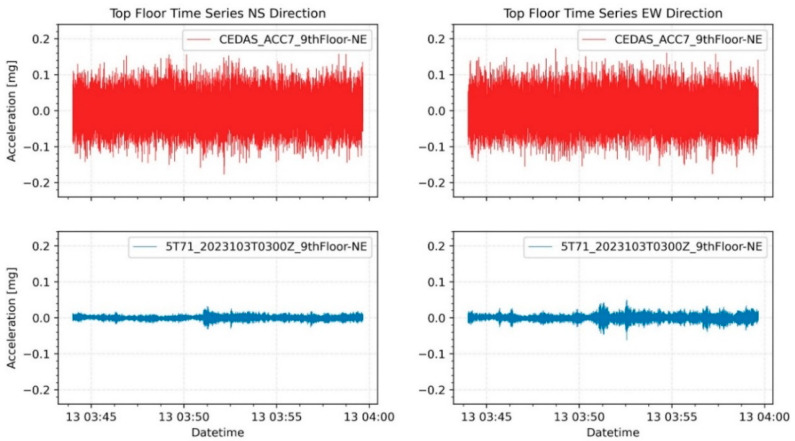
Representative ambient vibration time series of GURALP-5TDE and CEDAS devices from the 9th floor of the building in the NS and EW directions.

**Figure 23:**
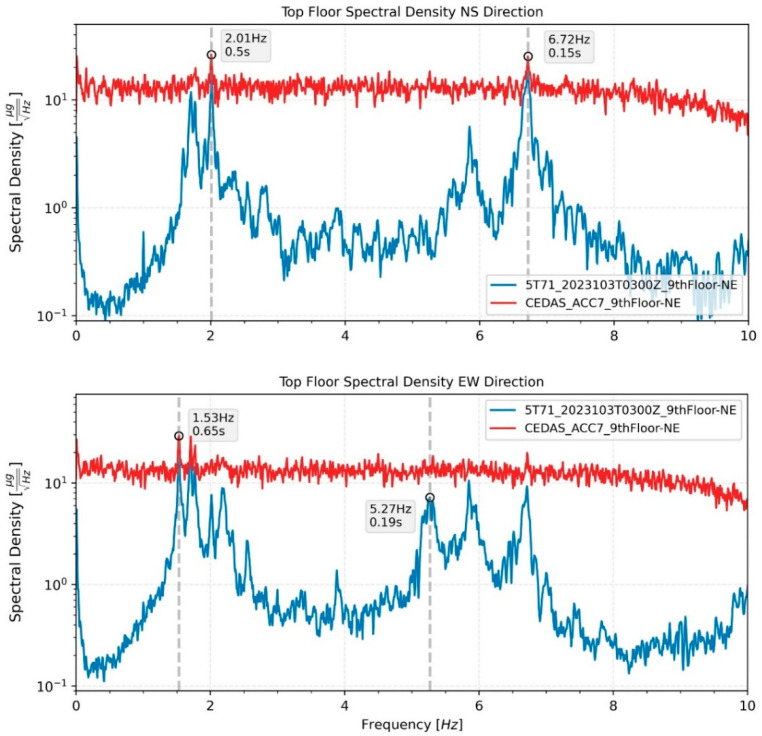
Spectral density and modal frequencies of representative ambient vibration data of Guralp and CEDAS devices from the top floor of the building in the NS and EW directions.

Ambient vibrations of the building were recorded from 11 April 2023, to 15 April 2023. The recordings were conducted between 18:00 PM and 12:00 AM, totaling approximately 18 h each day. A careful selection process was employed to choose five ambient vibration recordings with minimal contamination from aftershocks, ongoing construction work, traffic, and mechanical equipment noise. Representative time series of ambient vibration for both North-South (NS) and East-West (EW) directions are depicted in Figure 22. The analysis of these recordings was performed using the Spectral Density method. The first and second modal frequencies for devices situated on the 9th floor in the Northeast (NE) direction are illustrated in Figure 23 for both the NS and EW directions.

## 5. Conclusions and Future Studies

This study comprehensively reviewed low-cost sensor alternatives and developed an acceleration and velocity Data Acquisition System (DAS) utilizing open-source Python software, catering to applications in Earthquake Engineering and Earth Sciences. Validation tests and field experiments were conducted for two distinct CEDAS devices, utilizing a MEMS accelerometer sensor and analog geophone sensors, both running on a Raspberry Pi single-board mini-computer with network connectivity. The CEDAS devices, designed to compensate for each other’s limitations, offer a wide dynamic range when used in tandem.

The evaluation of CEDAS devices involved offset, frequency response, shake table, and spectral noise density tests. The offsets of CEDAS_acc devices were calibrated, and the sensitivity error was found to be lower than 1% for each axis, meeting the ANSS guidance for strong ground motion accelerometers. The frequency response of the CEDAS_geo device was assessed by calculating the transfer function with a GURALP-5TDE reference sensor, and the resulting response was flattened using a designed digital filter. CEDAS_acc was tested on a shake table alongside a GURALP-5TDE reference sensor, showing accurate timing and frequency response with overlapping signals. Spectral density estimates exhibited deviations of up to 1.5% compared to the GURALP-5TDE reference device, indicating a flat response within the −3 dB in-band limit for the tested frequency range of 0.5 Hz to 10 Hz. CEDAS_acc also demonstrated flat self-noise at −78 dB, or 15 μg. Meanwhile, CEDAS_geo exhibited noise density between low and high noise models [33], with further tests required for noise density validation under appropriate conditions.

The performance of CEDAS devices was assessed on a residential reinforced concrete structure, subjecting the building to both forced and ambient vibrations. Peak values derived from the forced vibration acceleration time series and modal frequencies calculated from spectral density estimates are closely aligned with those obtained using a GURALP-5TDE reference sensor. Table 3 provides details on the first and second modes for the building in both directions. Notably, CEDAS_acc exhibited time drifts on a scale of seconds, attributed to the internal clock of the MEMS sensor not operating at the center frequency. This phenomenon introduced time drifts and minor shifts in the frequency domain. However, it is crucial to highlight that this issue did not exert a significant impact on the obtained results.

The comparison of ambient and forced vibration data reveals slight changes in the natural frequencies of the buildings, indicating the linear behavior of the structure during seismic events. Field tests demonstrated that CEDAS_acc is proficient in forced vibration analysis for low-to mid-rise buildings. The modal frequencies obtained are well-correlated with the GURALP-5TDE reference sensor, and peak acceleration values deviated by a maximum of 1.5% for the building. However, when integrating to calculate velocity and displacement series, the impact of noise becomes more pronounced, causing waveform deviations and peak values to deviate from the reference device. At higher Signal-to-Noise Ratios (SNR), the velocity and displacement time series become less reliable. In contrast, CEDAS_geo offers significantly higher SNR, resulting in highly correlated peak acceleration, velocity, and displacement values with the GURALP-5TDE device. The summarized results for the building are presented in Table 4.

In future endeavors, the evolution and refinement of CEDAS devices could be directed towards the integration of advanced signal processing techniques, exploration of novel sensor technologies, and optimization of data storage and retrieval mechanisms to enhance overall performance and user-friendliness. External synchronization is a viable avenue for CEDAS devices. Full external synchronization or the utilization of interpolation methods can lead to more precise sampling rates and improved synchronization, particularly addressing issues such as minor time drift and frequency domain offsets, especially for the CEDAS_acc device [28]. To further enhance timing accuracy, employing a programming language faster than Python for sensors and ADC drivers could be considered. The implementation of a Real-Time Operating System (RTOS) or a real-time kernel for the Raspberry Pi computer may facilitate the execution of tasks and processes with heightened precision compared to the current operating system of the device. Additionally, CEDAS devices can potentially offer functionalities such as serving web pages for sensor readings, sending email alerts for specified conditions, and utilizing FTP (File Transfer Protocol) to transfer waveforms or analyzed results into databases or directly to clients. The incorporation of these features can be implemented within the RECANA web application.

## Figures and Tables

**Table 1:** SPI Connection of the CEDAS_geo ADS1256 ADC to Raspberry Pi.

Pin Description	ADS1256 Pin-Out	Raspberry Pi-4 Pin-Out
3.3 V Power	VCC	1
Ground	GND	6
Data Ready	DRDY	11
Reset	RST	12
Power Down	PDWN	13
Chip Select	CS	15
Master Out Slave In	DIN	19
Master In Slave Out	DOUT	21
Serial Clock	SCLK	23

**Table 2:** SPI connection of the CEDAS_acc ADXL355 sensor board to Raspberry Pi.

Pin Description	ADXL355 Pin-Out	Raspberry Pi-4 Pin-Out
3.3 V Digital Power	VDDIO (1)	1
3.3 V Digital Power	VDD (3)	1 or 17
Ground	GND (5)	9
Data Ready	DRDY (6)	11
Chip Select	CS (8)	24
Serial Clock	SCLK (10)	23
Master In Slave Out	MISO (11)	21
Master Out Slave In	MOSI (12)	19

**Table 3:** Shows the first and second mode periods of the building in both directions for forced vibration analysis.

		NS Direction		EW Direction	
		1st Mode	2nd Mode	1st Mode	2nd Mode
GURALP-5TDE	9th Floor	0.51	0.151	0.66	0.193
CEDAS_acc6	9th Floor	0.51	0.151	0.66	0.193

**Table 4:** Recorded acceleration, velocity, and displacement peak values of GURALP-5TDE and CEDAS_acc and CEDAS_geo devices from the building in both directions.

Device	Direction	Absolute Peak Values		
		Acceleration (mg)	Velocity (mm/s)	Displacement (mm)
GURALP-5TDE	NS	0.611	0.382	0.026
	EW	0.741	0.341	0.024
CEDAS_acc	NS	0.607	0.422	0.041
	EW	0.712	0.372	0.031

## Data Availability

Data will be shared when there is a demand.
